# Four new species of *Acalypha* L. (Euphorbiaceae, Acalyphoideae) from the West Indian Ocean Region

**DOI:** 10.3897/phytokeys.140.50229

**Published:** 2020-02-24

**Authors:** Iris Montero-Muñoz, Geoffrey A. Levin, José M. Cardiel

**Affiliations:** 1 Departamento de Biología, Facultad de Ciencias, Universidad Autónoma de Madrid. Ciudad Universitaria de Cantoblanco, Postal Code 28049, Madrid, Spain; 2 Centro de Investigación en Biodiversidad y Cambio Global (CIBC-UAM), Universidad Autónoma de Madrid. Ciudad Universitaria de Cantoblanco, Postal Code 28049, Madrid, Spain; 3 Canadian Museum of Nature, P.O. Box 3443, Station D, Ottawa, ON K1P 6P4, Canada; 4 Illinois Natural History Survey, Prairie Research Institute, University of Illinois, 1816 South Oak Street, Champaign, Illinois, 61820, United States of America

**Keywords:** Comoros, endemic, Madagascar, Mayotte, taxonomy

## Abstract

Four new species of *Acalypha* (Euphorbiaceae, Acalyphoideae) from the Western Indian Ocean Region, based on morphological and molecular evidence, are described, illustrated, and mapped. *Acalyphagillespieae* sp. nov., *A.leandrii* sp. nov. and *A.nusbaumeri* sp. nov. are endemic to Madagascar, and *A.mayottensis* sp. nov. is known only from Mbouzi islet (Mayotte), in the Comoros Archipelago. We also describe for the first time in *Acalypha* the presence of membranous or chartaceous perules covering the axillary buds. Preliminary conservation assessments of the new species are also provided.

## Introduction

This paper follows the revisionary work on *Acalypha* L. for the West Indian Ocean Region (Madagascar, the Comoros Archipelago, the Mascarene island, Scattered islands and Seychelles Archipelago) that was begun by Montero Muñoz et al. (2018a, b, in press).

*Acalypha*, with around 500 species, is the third largest genus in the family Euphorbiaceae, after *Euphorbia* L. (Riina et al. 2013) and *Croton* L. (Berry et al. 2005). It includes mainly small trees and shrubs of tropical and subtropical distribution, and some herbs that extend to temperate regions. About 65 species are found in continental Africa (Cardiel and Montero Muñoz 2018), and 39 species were previously known from West Indian Ocean Region (WIOR), most of them endemic there (Montero Muñoz et al. 2018a, b, 2020). A description of the main characteristics of the *Acalypha* species in WIOR, as well as the previous revisionary works in this region, can be found in Montero Muñoz et al. (2018a, b).

*Acalypha* is included in the subfamily Acalyphoideae, the most diverse in the Euphorbiaceae (Hayden and Hayden 2000). Although this subfamily is considered to be paraphyletic, the core group of included taxa (Acalyphoideae sensu stricto) is clearly monophyletic. Molecular analyses also support the monophyly of *Acalypha* (Tokuoka 2007, Wurdack and Davis 2009). Preliminary results of *Acalypha* molecular phylogeny (Levin et al. 2005, Sagun et al. 2010) suggest that the genus first evolved in Africa, where it is morphologically most diverse. Currently, we are working on the molecular phylogeny of *Acalypha* species from the WIOR (Montero Muñoz et al. in press) in the context of the phylogeny of the whole genus (Levin et al. in prep.). The preliminary results of this work also confirm the species here described as new. In all four newly described species, the axillary buds are covered by a pair of membranous or chartaceous perules; which are especially conspicuous in deciduous specimens. We have seen perules in other species of Acalypha from the WIOR region, usually associated with seasonally dry habitats. This is the first published report of the presence of perules in *Acalypha*.

## Materials and methods

The taxonomic status of the new species is based on morphological evidence, supported by geographical and ecological data. The descriptions and illustrations provided are based on field images and herbarium specimens located in CAN, G, HKM, ILLS, K, MAO, MO, P and TAN (abbreviations following Thiers 2020). Specimens seen by the authors are indicated with an exclamation mark (!). Herbarium barcodes are included when they are known. Specimens have been studied using a binocular microscope. Information about habit, plant size, and habitat are based on field notes on the specimen labels. The field photographs provided were made by the late Jean-Noël Labat and downloaded from the Muséum National d’Histoire Naturelle (Paris) website. The distribution maps were prepared using QGIS Desktop 3.2.2. Conservation assessments are based on the IUCN Red List Categories and Criteria (IUCN 2012, 2017). Area of occupancy (AOO) and extent of occurrence (EOO) were calculated with GeoCAT, a geospatial conservation assessment tool (Bachman et al. 2011; http://geocat.kew.org/), using a 2 × 2 km grid cell size as recommended by IUCN (2012, 2017).

All the taxonomic and biogeographical information about *Acalypha* is available online in the regularly updated “Acalypha Taxonomic Information System” website (Cardiel et al. 2020; www.acalypha.es).

## Taxonomic treatment

### 1. Acalypha gillespieae

Taxon classificationPlantaeMalpighialesEuphorbiaceae

G.A.Levin & I.Montero sp. nov.

2ED452A3-1E42-59D1-94B8-DD0296BDE496

urn:lsid:ipni.org:names:77206320-1

#### Diagnosis.

*Acalyphagillespieae* G.A.Levin & I.Montero is morphologically most similar to *A.humbertii* Leandri, but differs from it by having spherical axillary buds with imbricate perules (vs. pyriform buds with superposed perules), elliptic to obovate leaf blades (vs. ovate leaf blades), inflorescences c. 1 cm long with the fertile part of the male segment c. 1.5 mm long (vs. inflorescences c. 2.5 cm long with the male segment c. 20 mm), and mature female bracts subreniform with entire margins (vs. bracts suborbicular with dentate margins).

#### Type.

**Figure 1.**
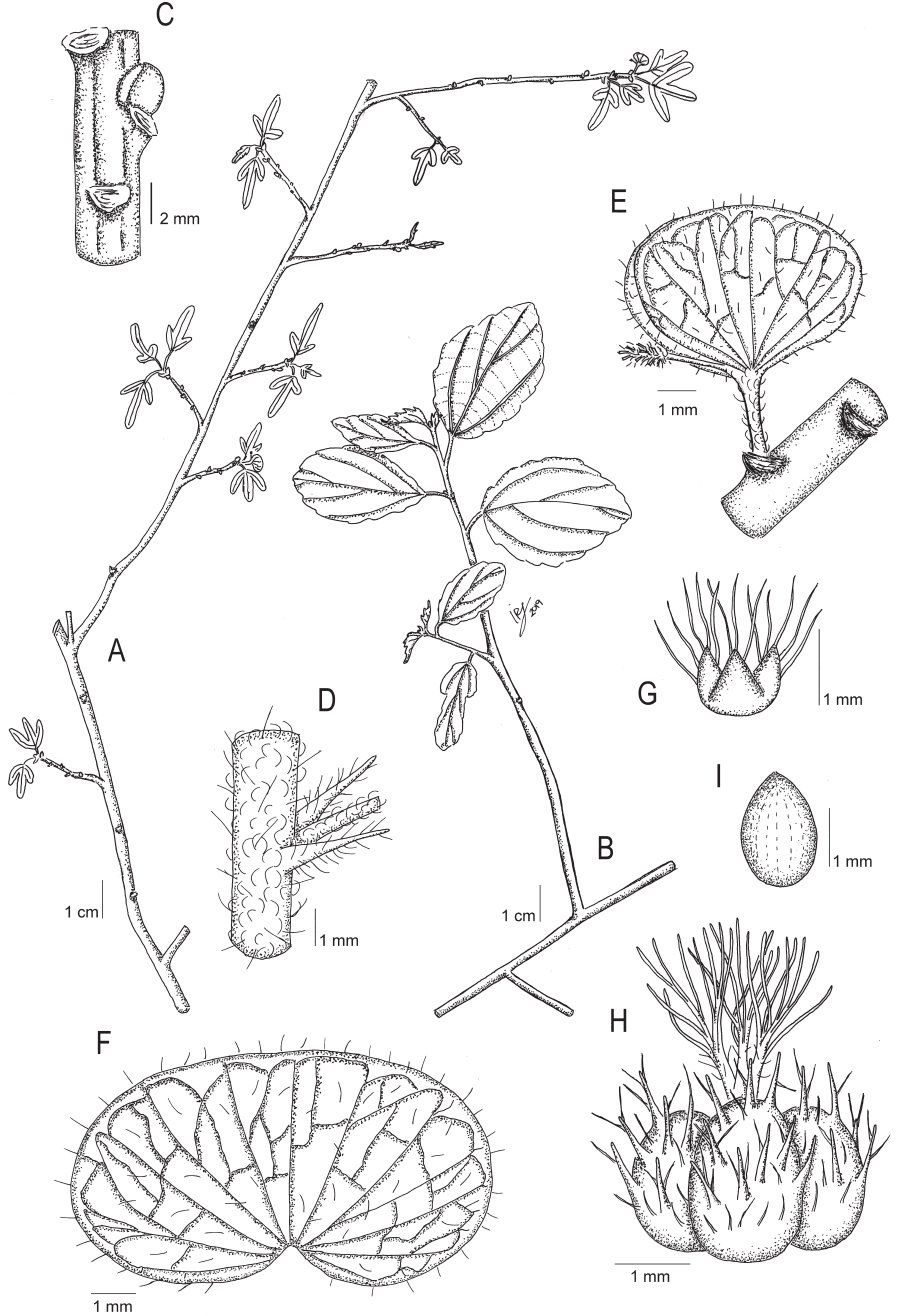
*Acalyphagillespieae***A** flowering branch with young leaves **B** flowering branch with mature leaves **C** detail of node with axillary bud **D** detail of node, stipules, and petiole base **E** detail of the androgynous inflorescence **F** mature female bract **G** calyx of the female flower **H** capsule with persistent styles **I** seed. Based on *L. Gillespie 4079* (**A, C, E**), *Gillespie et al. 10693* (**B, D**), and *Gillespie et al. 10692* (**F–I**). Illustration: Iris Montero Muñoz.

Madagascar. Reg. Diana [Prov. Antsirinana]: Montagne des Français, E of Antsirinana (Diego Suarez), 12°19'26.4"S, 49°20'16.6"E, 258 m, 31 Oct 2012, *L. J. Gillespie, G. A. Levin, J. Andriatiana, & W. M. Cardinal-McTeague 10692* (holotype: MO!; isotypes: CAN!, K!, P!, TAN!). Fig. 1.

#### Description.

***Shrubs*** to 3 m high, intricately branched, deciduous, monoecious. ***Young branches*** slender, densely pubescent with short, simple, straight, antrorsely appressed trichomes proximally, and antrorsely curved trichomes distally; older branches glabrescent. ***Axillary buds*** spherical, c. 2 mm diam., perules 2, imbricate, chartaceous, glabrous. ***Stipules*** deciduous, 2–3.5 mm long, subulate, densely pubescent with short, simple, spreading-ascending trichomes. ***Petioles*** slender, 2–5 mm long, densely pubescent with antrorsely curved trichomes. ***Leaf blades*** 1.5–4 × 1–3 cm, elliptic to obovate, membranous, unlobed or (2–)3-lobed; base rounded to broadly obtuse; margins crenate; apex obtuse; upper surface sparsely pubescent with simple, straight, erect to antrorse trichomes; lower surface with indumentum similar to that found on upper surface, but denser; venation actinodromous, somewhat prominent on both surfaces, with 3 veins at the base, secondary veins 2–3 per side. ***Stipels*** absent. ***Inflorescences*** androgynous, axillary, c. 1 cm long, spiciform, with one female bract near the base, and a male segment distally; peduncle thin, 2–3 mm long, densely pubescent with antrorsely curved trichomes; male segment persistent, sterile axis 1–2 mm, fertile portion c. 1.5 mm long, densely pubescent with simple, slender, flexuous trichomes. ***Female bracts*** sessile, enlarging in fruit to 5 × 9 mm, subreniform, sparsely pubescent with simple, straight, antrorse trichomes; margins entire. ***Bracteoles*** absent. ***Male flowers*** not seen (only the pedicels present). ***Female flowers*** solitary, sessile; sepals 3, slightly connate at base, c. 0.75 mm long, broadly triangular, ciliate with simple, slender, flexuous trichomes c. 0.5 mm long; ovary not seen; styles 3, persistent in fruit, c. 2 mm long, slightly connate at base, rachis stout, pubescent with short, simple, straight, antrorse trichomes, each style divided into 5–8 slender, fimbriate segments. ***Capsules*** 3-locular, c. 3 mm diam., echinate and hispid, with simple, straight, erect to antrorse trichomes c. 0.5 mm long, and conical projections c. 0.75 mm long. ***Seeds*** pyriform, 2 × 1.5 mm, smooth.

#### Distribution and habitat.

**Figure 2.**
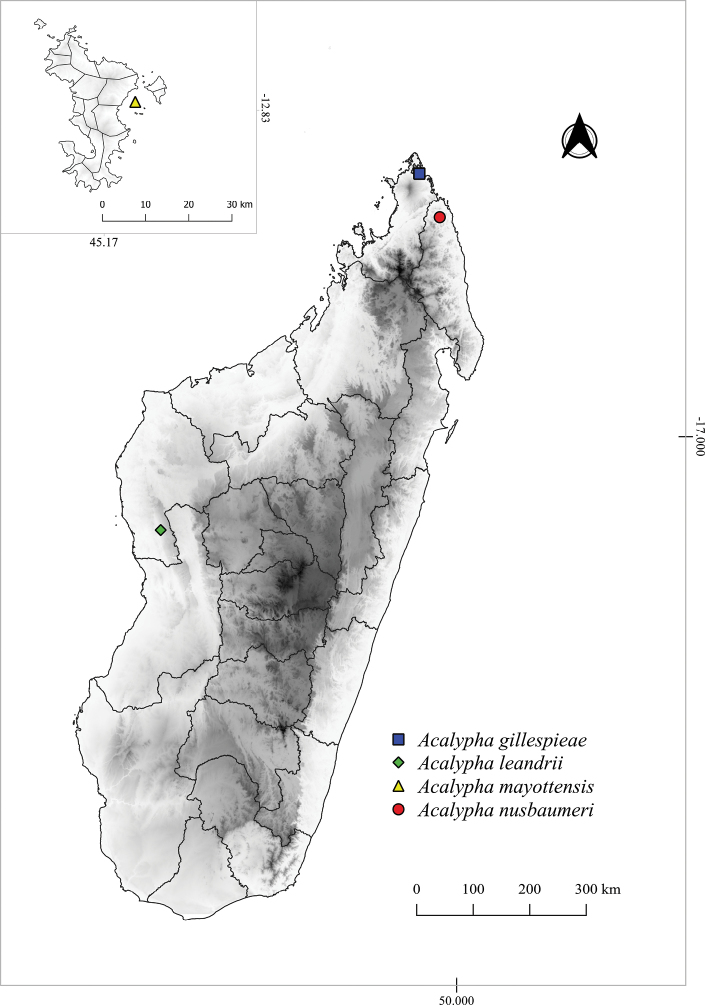
Map of Madagascar and the Mayotte island showing the distributions of *Acalyphagillespieae* (square), *A.leandrii* (rhombus), *A.mayottensis* (triangle) and *A.nusbaumeri* (circle).

*Acalyphagillespieae* is known only from a small area between 200 and 300 m elevation on the north side of Montagne des Français (Fig. 2). This limestone massif, including the area where *A.gillespieae* grows, is covered with dry deciduous forest (Moat and Smith 2007, Goodman et al. 2018).

#### Etymology.

The proposed epithet honors the botanist Lynn J. Gillespie, research scientist at the Canadian Museum of Nature. In addition to studying Arctic plants and Poaceae, she has worked on the systematics of Euphorbiaceae worldwide, including in Madagascar. She collected all the known specimens of this species, either alone or as leader of a team of botanists.

#### Conservation status.

*Acalyphagillespieae* is known from three collections from the same locality. Montagne des Français has been relatively well collected (P. Lowry, pers. comm.), so the dearth of collections suggests this species is rare, even there. Its apparent rarity could also, at least in part, reflect it being quite inconspicuous and thus easily overlooked. The extent of occurrence (EOO) could not be calculated. Its area of occupancy (AOO) is estimated to be 8000 km^2^. Montagne des Français is a category V protected area (Dudley 2008). Its habitat is somewhat threatened by wood-cutting, primarily for charcoal, but mainly on its lower slopes, below where *A.gillespieae* is found. *Acalyphagillespieae* is assigned a preliminary IUCN conservation status of Critically Endangered: CR B2ab(ii,iii,iv).

#### Additional specimens examined

**(paratypes).** Madagascar. Reg. Diana [Prov. Antsirinana]: Montagne des Français, E of Antsirinana (Diego Suarez), 12°19'26.4"S, 49°20'16.6"E, 258 m, 31 Oct 2012, *L. J. Gillespie*, *G. A. Levin*, *J. Andriatiana, & W. M. Cardinal-McTeague 10693* (CAN!, MO!, P!, TAN!); 12°19'S, 49°20'E, 200–300 m, 2 Dec 1990, *L. J. Gillespie 4097* (ILLS!, MO!, P[P00324524]!, TAN!).

#### Notes.

*Acalyphagillespieae* is very unusual among *Acalypha* species in having some lobed leaves. The proportion of lobed leaves varies among collections from about 10% in *Gillespie et al. 10693* to about 20% in *Gillespie et al. 10692* and 70% in *Gillespie 4097*. The lateral lobes range from much smaller than the central lobe to almost equal to it. The lobes, if present, arise near the base of the blade, with the basal veins becoming the midveins of the lobes. Like the very similar *Acalyphahumbertii*, *A.gillespieae* flowers when the plants are leafless, probably in late August or September. By the time the leaves emerge in late October, the male flowers have been shed and the female bracts and capsules are mature.

### 2. Acalypha leandrii

Taxon classificationPlantaeMalpighialesEuphorbiaceae

I.Montero & Cardiel sp. nov.

5ACC8E6F-7914-506F-A7D2-5CDE535D1964

urn:lsid:ipni.org:names:77206321-1

#### Diagnosis.

*Acalyphaleandrii* I.Montero & Cardiel is morphologically similar to *A.radula* Baker, but differs from it mainly by having leaf blades broadly ovate-lanceolate, not bullate, and petioles 2–8 cm long (vs. leaf blades usually narrowly triangular-lanceolate, bullate, and petioles 1.5–1.8 cm long), mature female bracts with entire margins (vs. mature female bracts with dentate margins), and capsules with simple trichomes (vs. capsules with simple and glandular trichomes).

#### Type.

**Figure 3.**
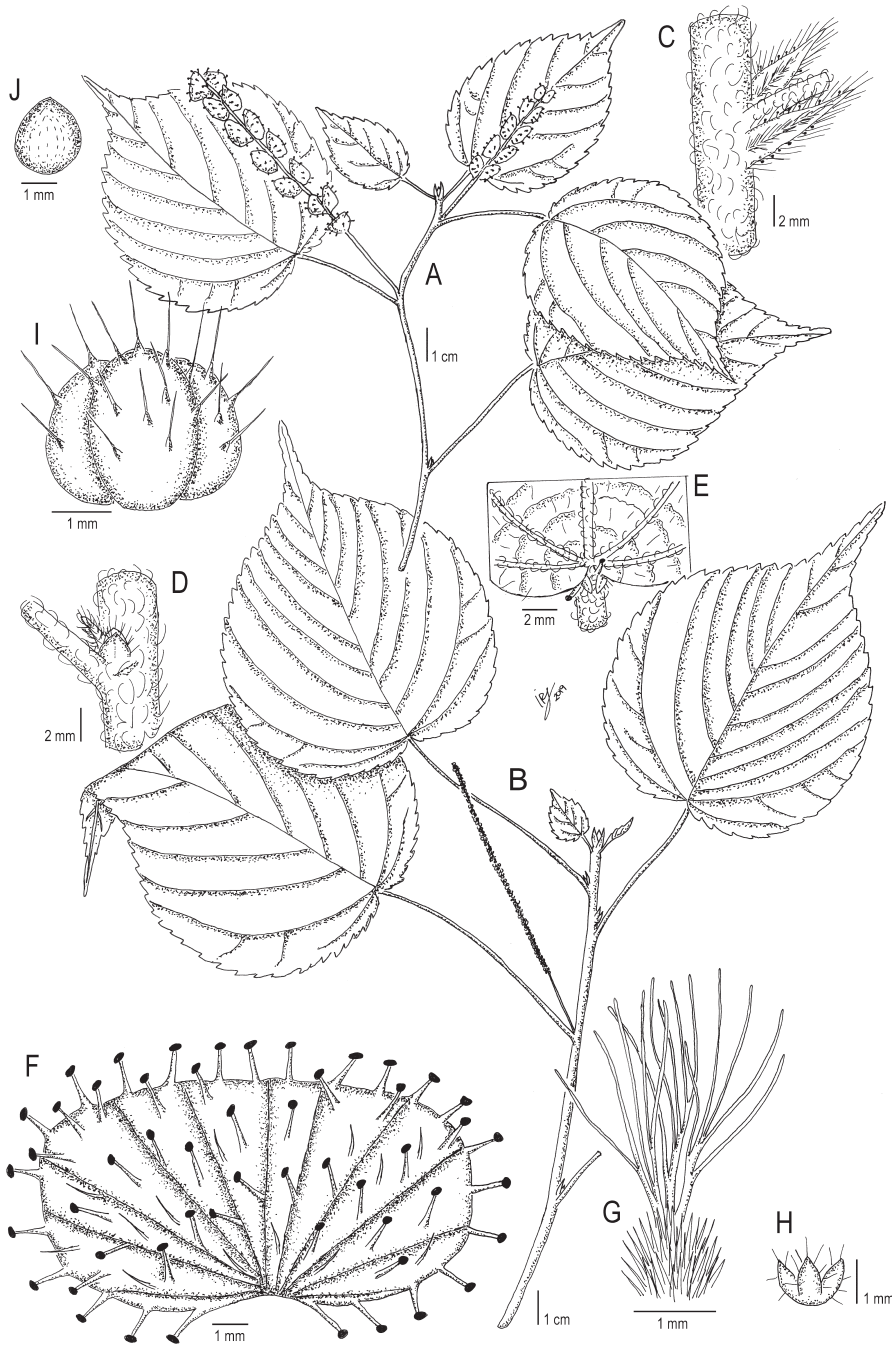
*Acalyphaleandrii***A** flowering female branch **B** flowering male branch **C** detail of node, stipules, and petiole base **D** detail of node with axillary bud **E** detail of upper leaf surface showing the leaf base and stipels **F** mature female bract **G** ovary and styles **H** calyx of the female flower **I** capsule **J** seed. Based on *J. Leandri, R. Capuron & A. Razafindrakoto 2037* (**A, C–J**) and *J. Leandri & P. Saboureau 2996* (**B**). Illustration by Iris Montero Muñoz.

Madagascar, Reg. Melaky [Prov. Mahajanga]: Antsalova, vers Ambodiriana (E. d’Antsalova), 18°40'0.12"S 44°43'59.879"E, 100–150 m, 06 Dec 1952, *J. Leandri, R. Capuron & A. Razafindrakoto 2037* (holotype: P [P05547059!]). Fig. 3.

#### Description.

***Shrubs*** or ***subshrubs*** (probably sprawling or clambering) evergreen [height unknown], probably dioecious. ***Young branches*** densely pubescent, with short, simple, antrorsely curved trichomes; older branches glabrous. ***Stipules*** c. 7 mm long, oblong-lanceolate, with scarious margins and a central rib; midrib appressed-pubescent, margins ciliate with thin trichomes mixed with minute glands. ***Axillary buds*** ovoid, c. 2 × 1 mm, perules 2, valvate, membranous, pubescent with short, simple trichomes. ***Petioles*** 2–8 cm long, indumentum similar to that found on the young branches, glabrescent. ***Leaf blades*** 8–12 × (3.5–) 4.5–9 cm, broadly ovate-lanceolate, membranous; base rounded to cordate; margins serrate, teeth acute, slightly callose-edged; apex acuminate to caudate, acumen acute, c. 2.5 cm long, mucronate; both surfaces laxly pubescent with simple, erect trichomes, also with short, antrorsely curved trichomes on veins; venation actinodromous, prominent in both surfaces, with 3 or 5 veins at the base, secondary veins 7–9 per side. ***Stipels*** triangular, c. 0.7 mm long, ciliate, mixed with glandular trichomes. ***Inflorescences*** unisexual, axillary, in terminal nodes. ***Male inflorescences*** spiciform, c. 8 cm long, peduncle c. 2 cm long, indumentum similar to that found on the young branches. ***Female inflorescences*** spiciform, with up to 18 bracts, c. 8.5 cm long, peduncle c. 2.5 cm long, indumentum similar to that found on the young branches. ***Female bracts*** sessile, enlarging in fruit to 6 × 12 mm, subreniform, with prominent veins on adaxial surface, laxly pubescent with erect, simple trichomes and thick glandular trichomes c. 1 mm long; margins entire. ***Male flowers*** inconspicuous, pedicel c. 0.5 mm long, sparsely hairy; buds c. 0.7 mm diameter, glabrous, papillose. ***Female flowers*** solitary, sessile; sepals 3, slightly connate at base, c.1 mm long, oblong-lanceolate, ciliate with simple, erect trichomes c. 0.5 mm long; ovary 3-locular, c. 1 mm diameter, densely hispid; styles 3, c. 5 mm long, slightly connate at base, each divided into 5 slender segments, glabrous. ***Capsules*** c. 3 mm diam., papillose-hispid, with papillae c. 0.5 mm long, each ending in a simple, erect trichome c. 1 mm long. ***Seeds*** pyriform, c. 2 × 1.6 mm, minutely foveolate.

#### Distribution and habitat.

*Acalyphaleandrii* is known from two localities in western Madagascar, in the area east of Antsalova. They are both from the karstic massif of Mesozoic limestones known as Tsingy de Bemaraha, in the Melaky Region. This region has dry climates, and the primary vegetation is dry deciduous forest (Schatz 2000, Moat and Smith 2007, Goodman et al. 2018). The altitudinal range of *A.leandrii* is from 100 to 300 m. (Fig. 2).

#### Etymology.

The proposed epithet honors the French botanist Jacques Désiré Leandri (1903–1982). He worked extensively in the Euphorbiaceae family from Madagascar, including writing the last taxonomic treatment of *Acalypha* from the island, in which he described numerous new species (Leandri 1942). Leandri collected the type specimen of this species.

#### Conservation status.

*Acalyphaleandrii* is known from three collections. The extent of occurrence (EOO) could not be calculated. Its area of occupancy (AOO) is estimated to be 8 km^2^. The Tsingy de Bemaraha lies within a national park and a nature reserve that has been IUCN category II and Ia protected areas (Dudley 2008, Goodman et al. 2018) since 1927 and a UNESCO World Heritage Site since 1990 (Goodman et al. 2018). The forest of this area has local anthropogenic pressures such as fire associated with the renewal of zebu (cattle) pastures, logging for construction and deforestation for new agricultural lands. Bemaraha has lost more forest habitat from 2006 to 2016 compared to 1996 to 2006 (Goodman et al. 2018). No specimens of this species have been collected for 60 years, so we cannot rule out that this species has become extirpated from one or both areas. In conclusion, due to habitat loss and the absence of recent collections, *A.leandrii* is assigned a preliminary IUCN conservation status of Critically Endangered: CR B2ab(ii,iii).

#### Additional specimen examined

**(paratypes)**. Madagascar. Reg. Melaky [Prov. Mahajanga]: Calcaires de l’Antsingy, vers Andobo (E. d’Antsalova), en remontant vers Tsiandro, 18°40'0.12"S, 44°43'59.879"E, 05–08 Feb 1960, 300 m, *J. Leandri & P. Saboureau 2996* (K!, P [P05543680!, P00324506!], MO [MO-3025001!], TAN); *J. Leandri & P. Saboureau 3016* (G!, P [P05547274!], MO [MO-2966304!]).

### 3. Acalypha mayottensis

Taxon classificationPlantaeMalpighialesEuphorbiaceae

I.Montero & Cardiel sp. nov.

49D883A8-9224-5723-9C0E-34CC15B678F7

urn:lsid:ipni.org:names:77206322-1

#### Diagnosis.

*Acalyphamayottensis* I.Montero & Cardiel is morphologically similar to *A.humbertii* Leandri, but differs from it mainly by having ovoid axillary buds with imbricate perules (vs. pyriform buds with superposed perules), triangular-lanceolate stipules c. 6 mm long (vs. linear stipules c. 3 mm long), and mature female bracts to 19 × 21 mm with crenate to subentire margins (vs. bracts to 6 × 8 mm with dentate margins).

#### Type.

**Figure 4.**
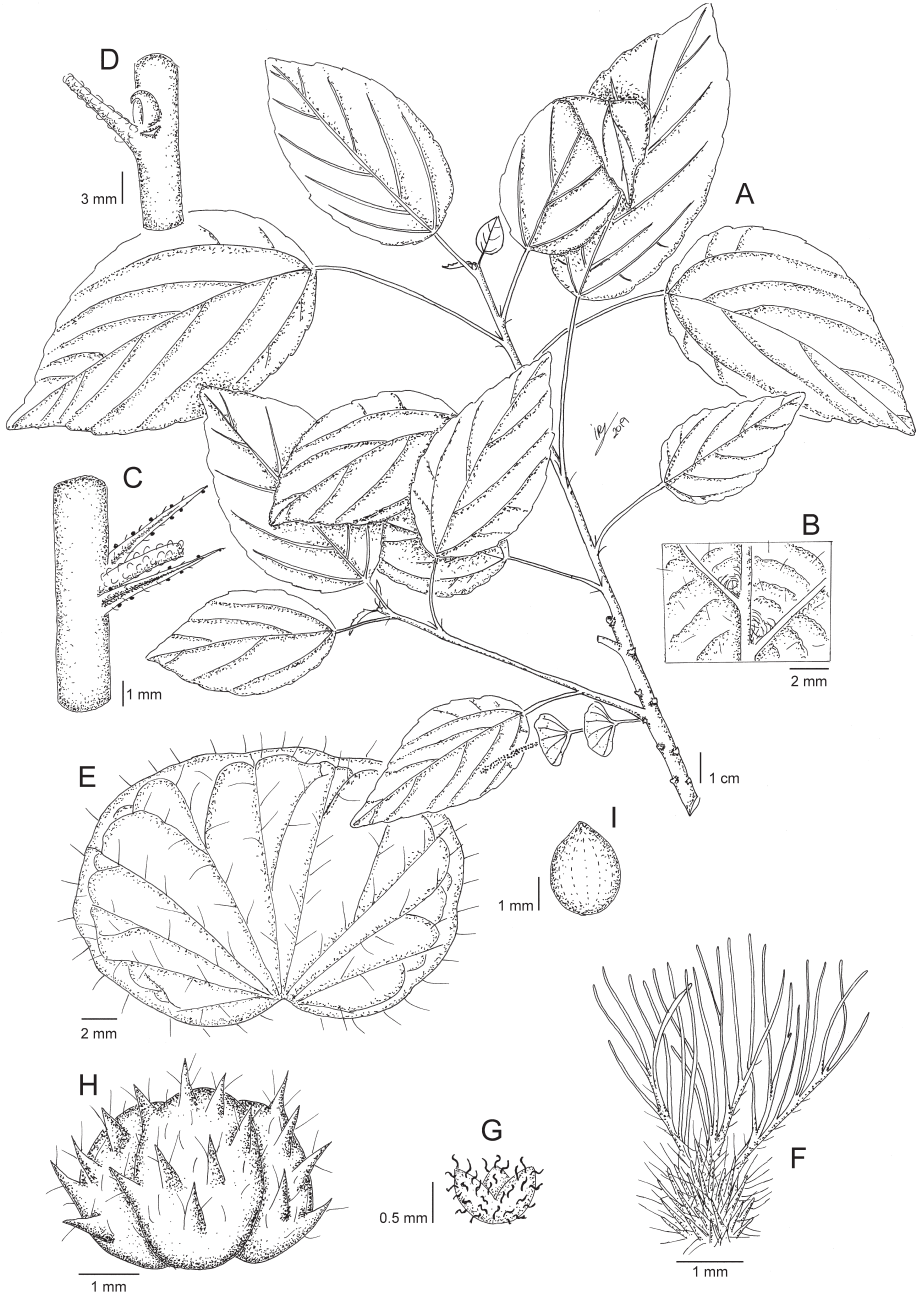
*Acalyphamayottensis***A** flowering branch **B** detail of lower leaf surface showing the domatia **C** detail of node, stipules, and petiole base **D** detail of node with axillary bud **E** mature female bract **F** ovary and styles **G** calyx of the female flower **H** capsule **I** seed. Based on *J.-N. Labat, F. Barthelat, C.M. Hladik & A.B. Sifary 3268* (**A–C**), and *J.-N. Labat, F. Barthelat, C.M. Hladik & A.B. Sifary 3272* (**D–I**). Illustration by Iris Montero Muñoz.

**Figure 5.**
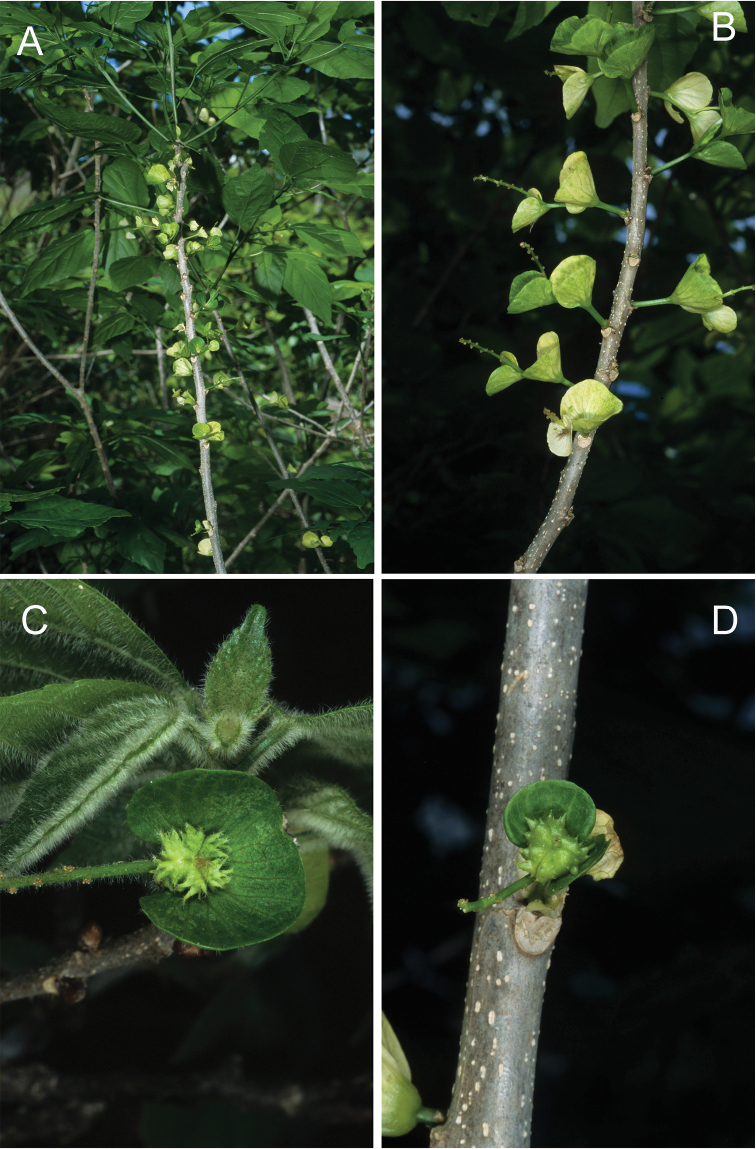
*Acalyphamayottensis***A** habit **B** flowering branch **C** female flower subtended by mature bract **D** capsule subtended by mature bract. Field images of type specimen. Photographs by Jean-Noël Labat.

Mayotte. Mamoudzou commune: Îlot M’bouzi, 12°48'50"S, 45°14'08"E, 10–50 m, 22 Nov 2000, *J.-N. Labat, F. Barthelat, C.M. Hladik & A.B. Sifary 3268*. (holotype: G [G00034240!]; isotypes: K!, MAO, MO [MO-2965774!], P [P00209719!]). Figs 4, 5.

#### Description.

***Shrubs*** to 5 m high, deciduous, monoecious. ***Young branches*** laxly pubescent, with simple, erect trichomes c. 1 mm long; ***Older branches*** glabrous. ***Axillary buds*** ovoid, c. 3 × 2.3 mm, perules 2, imbricate, blackish, chartaceous, glabrous. ***Stipules*** caducous, c. 6 mm long, linear to triangular-lanceolate, becoming filiform when mature, sparsely hairy, glabrescent, margins translucent, with some glands. ***Petioles*** slender, (2–) 3–5 (–6) cm long, pubescent with simple, antrorsely curved, trichomes. ***Leaf blades*** 5–10 × 3–6 cm, ovate-lanceolate to elliptic-lanceolate, membranous; base rounded to subcordate; margins crenate-serrate to subentire, slightly revolute, teeth minute, rounded, sinuses ciliate; apex subacuminate to acuminate, acumen c. 1.5 cm long, rounded; upper surface pubescent with simple, thin, patent, trichomes, glabrescent; lower surface with indumentum similar to that found on upper surface, but more dense; axils of the secondary veins with minute, sparsely hairy, pocket-shaped domatia, sometimes only hair-tuft domatia; venation actinodromous, with 3 veins at the base, secondary veins 4–6 per side. ***Stipels absent. Inflorescences*** androgynous, axillary, to 6 cm long, spiciform, with 1–2 female bracts near the base and a male segment distally; peduncle thick, c. 1.5 cm long, laxly pubescent, trichomes similar to those found on the young branches, glabrescent; male segment c. 4 cm long. ***Female bracts*** sessile, enlarging in fruit to 19 × 21 mm, subreniform, sparsely hairy with simple, erect trichomes c. 1.5 mm long on veins and margins, glabrescent; margins crenate to subentire, sometimes dentate in young bracts. ***Male flowers*** inconspicuous, pedicel c. 1 mm long, glabrous; buds c. 0.7 mm diameter, sparsely hairy, with arachnoid trichomes. ***Female flowers*** solitary, sessile; sepals 3[4], connate at base, c. 0.7 mm long, ovate-triangular, sparsely hairy with simple, arachnoid trichomes; ovary 3-locular, c. 1 mm diameter, echinate and hispid; styles 3, c. 3 mm long, free at the base, rachis thick, appressed-pubescent, each divided into 8–10 segments. ***Capsules*** to 4 mm diameter, echinate and hispid, with simple, erect trichomes c. 1 mm long, and conical projections c. 1 mm long, subacute. ***Seeds pyriform***, 2.5 × 2 mm, minutely foveolate.

#### Distribution and habitat.

*Acalyphamayottensis* is endemic to Mayotte, a French overseas department in the Comoros Archipelago, and presumably restricted to the Mbouzi islet (Fig. 2). Mbouzi is a small, volcanic, unoccupied islet, of 82 ha, located east of the main island (Grande-Terre). It has a tropical humid climate, with two seasons: one cool and dry, the other hot and wet, resulting from shifts in the Intertropical Convergence Zone. Mbouzi is mainly covered by secondary dry deciduous forest (Boullet and Traclet 2018). According to the specimens’ labels, *A.mayottensis* is a common deciduous bush on the islet, growing in deciduous forest, in ravines and stony areas, from 10 to 90 m elevation.

#### Etymology.

The proposed epithet refers to Mayotte island, to which the small Mbouzi islet belongs.

#### Conservation status.

*Acalyphamayottensis* is only known from Mbouzi islet. The extent of occurrence (EOO) is estimated to be 0.017 km^2^. Its area of occupancy (AOO) is estimated to be 8 km^2^. Mbouzi islet was declared a “Réserve Naturelle Nationale” in 2007, a category IV protected area (Dudley 2008). In the 1990s the islet had lost 70% of its original forests due to agricultural activities. Mbouzi currently conserves 10% of its natural and subnatural forest (Boullet and Traclet 2018). Currently, the most serious threat is invasive species, both animals, such as *Eulemurfulvus*, and plants, such as *Antigononleptopus*, *Lantanastrigocamara*, *Leucaenaleucocephala*, *Litseaglutinosa*, *Spathodeacampanulata* and *Furcraeafoetida* (Boullet and Traclet 2018, Quintard et al. 2019). *A.mayottensis* is assigned a preliminary IUCN conservation status of Critically Endangered: CR B1ab(i,iii) + B2ab(ii,iii).

#### Additional specimen examined

**(paratypes).** Mayotte. Mamoudzou commune: Îlot M’Bouzi, 12°48'57"S, 45°14'06"E, 90 m, 22 Nov 2000, *J.-N. Labat, F. Barthelat, C.M. Hladik & A.B. Sifary 3272* (G [G00034255!], K!, MAO, MO [MO-2966248!], P [P00209724!, P00209725!]); Îlot M’Bouzi, 12°48'39"S, 45°14'06"E, 26 Dec 2002, *F. Barthelat, A. de Vanssay & G. Rembert 1112* (MAO, P [P00339165!]); precise location unknown, probably from M’Bouzi islet, 01 Jan 2010, *G. Viscardi 310* (HKM, P [P02439826!]).

#### Notes.

Five other species of *Acalypha* are known from Mayotte: *Acalyphachibomboa* Baill., *A.indica* L., *A.lanceolata* (Müll.Arg.) Radcl.-Sm., *A.paxii* Aug.D.C., and *A.richardiana* Baill. *A.mayottensis* does not strongly resemble any of them. The only other *Acalypha* species known from Mbouzi islet is *A.richardiana*, which differs mainly by having sessile androgynous inflorescences and mature female bracts subrounded, c. 7 × 6 mm (vs. pedunculate androgynous inflorescences and mature female bracts subreniform, c. 19 × 21 mm in *A.mayottensis*). The herbarium specimens of *A.mayottensis* had been previously identified as *A.claoxyloides* Hutch., endemic to the Seychelles Archipelago, but it clearly differs by having flattened resinous glands on lower leaf surface, female bracts and flowers, and smooth capsules (vs. resinous glands absent and echinate capsules in *A.mayottensis*).

### 4. Acalypha nusbaumeri

Taxon classificationPlantaeMalpighialesEuphorbiaceae

I.Montero & Cardiel sp. nov.

6A5980B2-0EAB-5813-B8C6-BEE41AEF0A4E

urn:lsid:ipni.org:names:77206323-1

#### Diagnosis.

*Acalyphanusbaumeri* I.Montero & Cardiel is morphologically similar to *A.perrierii* Leandri, but differs from it mainly by having leaf blades with subacuminate apices (vs. leaf blades with caudate apices), inflorescences to 1.7 cm long (vs. inflorescences to 3 cm long), and mature female bracts 2 × 2.5 mm, translucent, with crenate margins and two basal bracteoles (vs. bracts 7 × 12 mm, opaque, with entire margins and no bracteoles).

#### Type.

**Figure 6.**
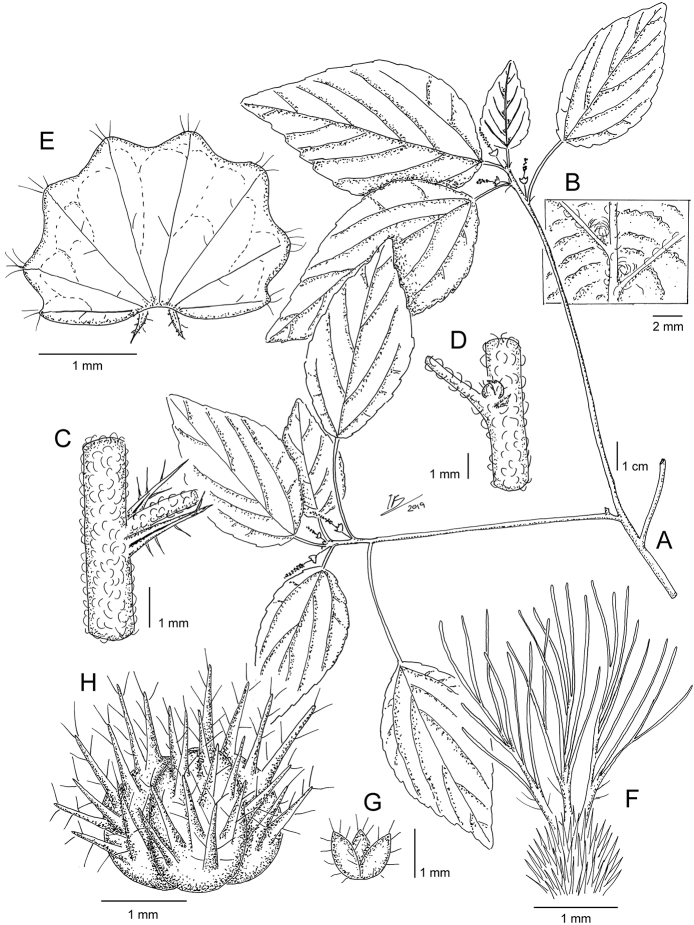
*Acalyphanusbaumeri***A** flowering branch **B** detail of lower leaf surface showing the pocket-shaped domatia **C** detail of node, stipules, and petiole base **D** detail of node with axillary bud **E** mature female bract with bracteoles **F** ovary and styles **G** calyx of the female flower **H** capsule. Based on *L. Nusbaumer & P. Ranirison LN1169*. Illustration by Iris Montero Muñoz.

Madagascar. Reg. Sava [Prov. Antsiranana]: sous-préfecture de Vohemar. Commune rurale de Daraina, forêt de Bekaraoka, partie nord, 13°06'S, 49°42'E, 177 m, 13 Feb 2004, *L. Nusbaumer & P. Ranirison LN1169.* (holotype: G [G00028080!]; isotypes: K!, MO!, P [P05547228!, P01152829!]). Fig. 6.

#### Description.

***Shrubs*** to 0.8 m high, probably deciduous, monoecious. ***Young branches*** very slender, divaricate, blackish, pubescent with simple, antrorsely curved trichomes; older branches glabrous. ***Axillary buds*** spherical, c. 1 mm diameter, perules 2, imbricate, membranous, external perule dentate, sparsely hairy. ***Stipules*** caducous, c. 2 mm, triangular-lanceolate, with a prominent central rib, sparsely hairy with simple, short, erect trichomes. ***Petioles*** slender, 2–3.5 (–4.5) cm long, with indumentum similar to that found on young branches. ***Leaf blades*** 5–8.5 × 2.5–5 cm, ovate-lanceolate to elliptic-lanceolate, thin-membranous; base obtuse to rounded; margins crenate-serrate; teeth rounded; apex subacuminate, acumen c. 0.5 mm long, rounded and mucronate at apex; upper surface subglabrous, with simple, short, antrorsly curved trichomes on veins; lower surface with indumentum similar to that found on upper surface, axils of the secondary veins with pocket-shaped, sparsely hairy domatia; venation actinodromous, with 3 or 5 veins at the base, secondary veins 3–4 per side. ***Stipels*** absent**. *Inflorescences*** inconspicuous, androgynous, axillary, c. 1.7 cm long, spiciform, mostly male with 1 female bract near the base; peduncle thick, c. 0.5 cm long, with indumentum similar to that found on the young branches; male segment c. 7 cm long. ***Female bracts*** sessile, translucent, enlarging in fruit to 2 × 2.5 mm, orbicular-reniform, subglabrous, with short, simple trichomes on teeth and veins, margins crenate. ***Bracteoles*** linear-lanceolate, c. 0.5 mm long, sparsely hairy, with short, simple trichomes, and some sessile glands on the margins. ***Male flowers*** inconspicuous, pedicel c. 0.5 mm long, sparsely hairy; buds c. 0.8 mm diameter, glabrous, papillose. ***Female flowers*** solitary, sessile; sepals 3, free at base, c. 1 mm long, lanceolate, ciliate with short, simple trichomes; ovary 3-locular, c. 1 mm diameter, densely hispid; styles 3, c. 3.5 mm long, free at the base, each divided into 8–10 slender segments, rachis sparsely hairy. ***Capsules*** (immature) to 2 mm diam., echinate, with projections c. 1 mm long, pubescent with simple, short erect trichomes c. 0.5 mm long. ***Seeds*** too young to describe.

#### Distribution and habitat.

*Acalyphanusbaumeri* is only known from Bekaraoka forest, in the Loky-Manambato protected area, in Sava Region, northern Madagascar (Fig. 2). This area has a seasonally dry climate (Schatz 2000; Goodman et al. 2018). Regarding its vegetation, Loky-Manambato is a special area because it is between the Eastern Humid and Western Dry phytogeographic domains and so has many types of vegetation (Nusbaumer et al. 2010). Bekaraoka forest has dry deciduous forest on basement rocks (Moat and Smith 2007, Goodman et al. 2018), which seems to be the characteristic habitat of *A.nusbaumeri*.

#### Etymology.

The proposed epithet honors Louis Nusbaumer, researcher and curator of Conservatoire et Jardin botaniques de la Ville de Genève, Switzerland. He works on the systematics, phylogeny, biogeography, and conservation of Malagasy plants. Nusbaumer is also the collector, with Patrick Ranirison, of the type specimen of this species.

#### Conservation status.

*Acalyphanusbaumeri* is only known from one collection. The extent of occurrence (EOO) could not be calculated. Its area of occupancy (AOO) is estimated to be 8 km^2^. Loky-Manambato is a category V (Goodman et al. 2018) protected area since 2005. The forest in this region has been degraded and continues to be threatened by slash and burn agriculture, fires to clear land for grazing, illegal cutting of precious woods, and in some areas, as Bekaraoka, gold extraction (Vargas et al. 2002, Rakotondravony 2009, Goodman et al. 2018). *Acalyphanusbaumeri* is assigned a preliminary IUCN conservation status of Critically Endangered: CR B2ab(ii,iii).

## Supplementary Material

XML Treatment for Acalypha gillespieae

XML Treatment for Acalypha leandrii

XML Treatment for Acalypha mayottensis

XML Treatment for Acalypha nusbaumeri
